# Serum activity of DPPIV and its expression on lymphocytes in patients with melanoma and in people with vitiligo

**DOI:** 10.1186/1471-2172-13-48

**Published:** 2012-08-21

**Authors:** Ivana Z Matić, Marija Đorđić, Nađa Grozdanić, Ana Damjanović, Branka Kolundžija, Aleksandra Erić-Nikolić, Radan Džodić, Miomir Šašić, Srđan Nikolić, Danijela Dobrosavljević, Sanvila Rašković, Slađana Andrejević, Dušica Gavrilović, Oscar J Cordero, Zorica D Juranić

**Affiliations:** 1 Institute of Oncology and Radiology of Serbia, Pasterova 14, 11000, Belgrade, Serbia; 2 School of Medicine, University of Belgrade, 11000, Belgrade, Serbia; 3 Clinic of Dermatovenereology, Clinical Center of Serbia, 11000, Belgrade, Serbia; 4 Institute of Immunology and Allergology, Clinical Center of Serbia, 11000, Belgrade, Serbia; 5 Department of Biochemistry and Molecular Biology, University of Santiago de Compostela, CIBUS Building, r/Lopez de Marzoa s/n, Campus Vida, 15782, Santiago de Compostela, Spain

**Keywords:** CD26 expression, DPPIV serum activity, Melanoma, Vitiligo

## Abstract

**Background:**

Dipeptidyl peptidase IV, a multifunctional serine protease, is implicated in regulation of malignant transformation, promotion and further progression of cancer, exerting tumor-suppressing or even completely opposite - tumor-promoting activities.

The aim of present research was to determine the serum DPPIV activity, as well as the percentages of CD26+ lymphocytes, CD26+ overall white blood cells and the mean fluorescence intensity of CD26 expression on lymphocytes in patients with melanoma, people with vitiligo and in healthy controls.

**Methods:**

The activity of DPPIV in serum was determined by colorimetric test. Expression of DPPIV (as CD26) on immunocompetent peripheral white blood cells was done using flow cytometry analysis.

**Results:**

Data from our study show for the first time statistically significant decrease: in the serum DPPIV activity, in the percentage of CD26+ overall white blood cells and in the percentage of lymphocytes in patients with melanoma in comparison to healthy control people. In addition, significantly lower serum DPPIV activity was found in the group of patients with melanoma in relation to people with vitiligo too.

**Conclusion:**

This study indicates the need for exploring the cause and the importance of the disturbances in the serum DPPIV activity and in the CD26 expression on immunocompetent cells in complex molecular mechanisms underlying the development and progression of melanoma.

## Background

Elucidation of molecular mechanisms involved in carcinogenesis and understanding the complex crosstalk between immunity and cancer represent one of the crucial steps in development of novel, better approaches in prevention, diagnosis and therapy of malignant diseases. Many recent studies focused on biology of cancer indicate the prominent role of dipeptidyl peptidase IV (DPPIV or CD26) in initial steps of malignant transformation, promotion and progression of tumors, acting as a tumor suppressor or even tumor activator. Moreover, DPPIV is implicated in the control of diverse biological processes, especially immune functions and inflammation [1-4].

By cleaving the dipeptides from N- terminal end of peptides and polypeptides that have proline or alanine in the second position, DPPIV controls the activity of many bioactive molecules, including cytokines and chemokines, incretins and gastrointestinal hormones, vasoactive peptides and neuropeptides [1-3]. Besides its enzymatic activity, DPPIV acts as receptor for plasminogen type 2 and adenosine deaminase (ADA), interacts with chemokine receptor CXCR4 and with mannose 6-phosphate/insulin-like growth factor II receptor. Furthermore, DPPIV exerts costimulatory function by association with tyrosine phosphatase CD45. Interaction with proteins, components of extracellular matrix, such as collagen and fibronectin, points to its role in adhesion, invasion and metastasis of cancer cells [1-3,5]. Its involvement in regulation of apoptosis has been reported too [1] and references cited therein.

Data from many reports show association of altered CD26 expression levels on the cell surface as well as changes in DPPIV/CD26 activity or sCD26 levels, with various types of cancer [1-12]. Tumor-suppressing activity of DPPIV is supported by facts that decrease and loss of DPPIV expression and activity are found in microenvironments of specific tumors and also in systemic circulation [2]. Decrease in DPPIV is shown in melanoma, prostatic, endometrial, colorectal, hematological and renal cancers, and in lung and skin squamous cell carcinomas. On the contrary, elevated DPPIV expression and/or activity are related to thyroid follicular carcinoma, astrocytic tumors, gastrointestinal stromal tumors, T and B lymphomas and leukemias, revealing the tumor-promoting activities of this molecule, as reviewed by Cordero et al. [2] and references cited therein. It is very important to note that lower serum DPPIV enzymatic activity, related with impaired immune functions, is found in patients with various types of hematological malignancies and different solid malignant tumors, including oral cancer, gastric carcinoma, colorectal cancer and thyroid cancer. Increase in serum DPPIV activity, associated with enhanced immunity, occurs in hepatic cancer and in some hematological neoplasmas [2] and references cited therein.

Decreased expression of CD26 during malignant transformation of melanocytes and even the absence of this molecule on primary and metastatic melanoma cells is already proved [13-17], but still there are no data on the extent of the expression of this molecule on immunocompetent cells and its serum activity in melanoma patients. These missing facts about DPPIV, implicated in regulation of tumorigenesis and immune functions, could be significant especially because melanoma is highly immunogenic malignant tumor [18]. On the other hand, it is documented that 10% of patients with melanoma develop hypopigmentation of the skin that resembles vitiligo, an autoimmune dermatological disorder. In addition, enhanced antitumor immune response observed in mentioned melanoma patients is considered to be connected with a better prognosis [18,19].

Assessment of changes in the DPPIV activity in patient's sera and in the percentages of CD26+ immunocompetent cells was done with the aim to clarify the possible role of DPPIV in development and progression of melanoma. Regarding the possible connection between melanoma and vitiligo, it would be important to examine if there are any changes in DPPIV expression levels on lymphocytes and serum activity in people with vitiligo.

## Methods

### Patients

This research involved 64 patients with melanoma (60.48 ± 14.81 (mean age ± SD), age range 23–86 years; 34 males and 30 females) and 16 people with vitiligo (47.62 ± 11.56 (mean age ± SD), age range 26–64 years; 5 males and 11 females). In addition, 6 patients with other malignant skin tumors (64.83 ± 14.34 (mean age ± SD), age range 37–75 years; 4 males and 2 females) and 6 patients with benign skin changes (53.50 ± 22.92 (mean age ± SD), age range 16–75 years; 1 male and 5 females) were also included in the study. Samples were obtained before surgical resection of the tumor. It should be noted that 37 melanoma patients were with metastatic disease. The control group consisted of 40 apparently healthy donors (39.13 ± 10.65 (mean age ± SD), age range 24–62 years; 14 males and 26 females). The protocol of the study was approved by the Ethics Committee of the Institute of Oncology and Radiology of Serbia. Written informed consent was obtained from each patient.

### DPPIV activity in serum

The activity of DPPIV in patient’s serum was determined by direct photometric method adapted to 96-well microtiter plates and according to the procedure described by Jarmolowska et al. [20], but modified to some extent. In serum samples and their blanks were added 50 μL of 0.3 M Gly/NaOH buffer (pH 8.7), 20 μL of 1.5 mM Gly-Pro-*p*-nitroanilide *p*-toluensulfonate (DPPIV substrate, Sigma G2901) and 50 μL of distilled water. Samples of standard contained 20 μL of 1.5 mM *p*-nitroaniline (Sigma N2128) instead of substrate and their blanks contained 20 μL of water. After 30 min of incubation at 37°C, 50 μL of chilled (4°C) 1 M acetate buffer (pH 4.2), was added to blanks of serum samples in order to prevent the enzymatic reaction. Then 10 μL of serum was added to serum samples and their blanks. In standard samples and standard’s blanks 10 μL of water was added. Plates were incubated for another 30 min at 37°C. Enzymatic reaction was stopped by adding 50 μL of chilled (4°C) 1 M acetate buffer (pH 4.2) in samples that contained serum. Samples of standard and their blanks also contained the same volume of acetate buffer. The absorbance was measured at 405 nm. DPPIV activity in serum was calculated according to the formula:

(1) $$\begin{array}{l} \text{Enzyme activity}=\left(\text{A serum sample}-\text{A blank}\text{ of serum }\text{sample}/\text{A standard}-\text{A blank of standard}\right)*100\\ \text{Enzyme activity}:\;\text{IU}/\text{L}=1\;\mu \text{M}/\text{min}/\text{L of serum} \end{array}$$

All experiments were done in triplicate. Cut-off values were (Xav ± SD) IU/L.

### Flow cytometry analysis

Expression levels of CD26 on white blood cells were evaluated by flow cytometry analysis. Briefly, 50 μL of whole blood was added to 2.5 μL (0.05 mg/mL) of solution of Ab, reaction mixture was vortexed, and incubated in the dark for 15 min. Then 800 μL of lysing solution was added, vortexed and incubated in the dark for 10 min, centrifuged for 5 min at 1600 rpm. Cell samples were washed two times and at the end 200 μL of cell fix or cell wash solution was added. Monoclonal antibody specific for CD26 was PE-stained (Becton Dickinson Immunocytometry Systems, CA, USA, catalog number 340423). Expression of CD26 on cells, observed mostly on lymphocytes, (sorted by physical gating) was determined using a FACSCalibur flow cytometer (BD Biosciences Franklin Lakes, NJ, USA). Acquired data were analyzed using CELLQuest Software (BD Biosciences). Obtained data were presented not only as the expression on lymphocytes, but also additionally as percentage of CD26 expression on total white blood cell pool (lymphocytes, monocytes and granulocytes). Cut-off values for analyzed parameters were (Xav ± SD).

### Statistical methods

Statistical significance of differences in studied parameters between examined groups was estimated using Fisher exact test, Kruskal Wallis test, Wilcoxon rank sum test and Wilcoxon rank sum test with continuity correction. Normal distributions of parameters were tested using the Kolmogorov-Smirnov test and Shapiro-Wilk test. For analyses was used statistical programme R version 2.14.1 (2011-12-22) Copyright (C) 2011 The R Foundation for Statistical Computing, ISBN 3-900051-07-0. Bonferroni correction used for the determination of the statistical significance of results obtained, set up the value of (p < 0.05/10) as the limit.

An abnormal elevation of the value obtained for each analyzed parameter was defined as any value above the mean value for the control healthy people group plus the standard deviation and an abnormal reduction as any value below the mean value for the control group minus the standard deviation.

## Results

### Activity of DPPIV in serum

**Figure 1:**
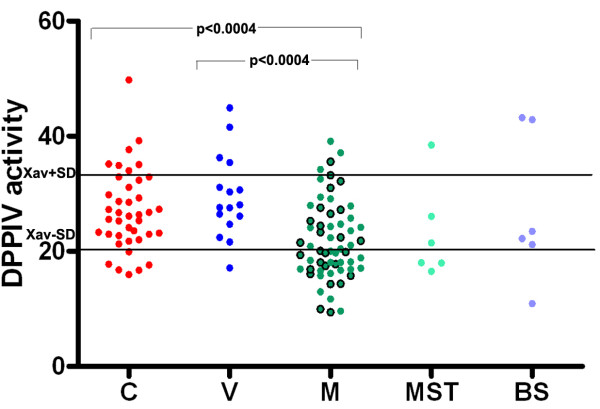
**Serum DPPIV activity in healthy controls (C), in people with vitiligo (V), in patients with melanoma (M), and in patients with other malignant skin tumors (MST) or benign skin changes (BS).** Xav+/−SD are cut off values obtained from healthy controls. Green circles with borders represent melanoma patients without metastases, while green circles without borders represent melanoma patients with metastatic disease. There is a statistically significant decrease in the DPPIV serum activity in patients with melanoma in comparison to healthy controls (Wilcoxon rank sum test with continuity correction: W = 723; p < 0.0004) and as well as in relation to people with vitiligo (Wilcoxon rank sum test with continuity correction: W = 206; p < 0.0004).

Data from the present research show that there is a statistically significant decrease in the serum DPPIV activity in patients with melanoma in comparison to healthy controls (p < 0.0004), as well as in comparison to people with vitiligo (p < 0.0004). These results are presented in Table 1 and on Figure 1. There was no difference in the obtained values for DPPIV activity in serum between vitiligo and healthy control groups. Lower serum DPPIV activity was also found in patients with other malignant skin tumors compared to people with vitiligo and to healthy controls.

**Table 1:** Activity of DPPIV in serum, % of CD26+ lymphocytes and % of CD26+ white blood cells, mean fluorescence intensity (MFI) of CD26 expression on lymphocytes and % of lymphocytes in healthy controls, people with vitiligo, patients with melanoma, and in patients with other malignant skin tumors and benign changes of the skin

**Patients**	**n**	**DPPIV serum activity (IU/L)**	**% of CD26+ lymphocytes**	**% of CD26+ white blood cells**	**MFI of CD26 expression on lymphocytes**	**% of lymphocytes**
**Healthy controls**	40	27.03 ± 7.00	50.33 ± 11.85	11.26 ± 5.26	278.41 ± 138.82	20.65 ± 8.48
**Vitiligo**	16	29.51 ± 7.24	48.04 ± 10.02	8.41 ± 3.30	690.29 ± 907.96	16.50 ± 4.51
**Melanoma**	64	21.81 ± 6.79 *	52.93 ± 10.77	7.16 ± 3.63 **	377.21 ± 554.38	13.43 ± 6.13 ***
**Melanoma without metastatic disease**	27	21.53 ± 6.74	53.24 ± 9.92	6.40 ± 3.28	409.51 ± 686.61	12.18 ± 5.91
**Melanoma with metastatic disease**	37	22.02 ± 6.91	52.71 ± 11.49	7.71 ± 3.81	353.64 ± 442.73	14.35 ± 6.19
**Other malignant skin tumors**	6	23.08 ± 8.28	47.17 ± 7.93	7.06 ± 3.28	235.73 ± 172.32	13.36 ± 6.02
**Benign skin changes**	6	27.32 ± 12.96	54.64 ± 10.78	9.19 ± 4.32	249.45 ± 237.82	15.77 ± 6.17

* p <0.0004 in comparison to healthy controls and to people with vitiligo.

** p < 0.00006 in comparison to healthy controls.

***p < 0.00003 in comparison to healthy controls.

It should be noted that 30 out of 62 analyzed patients with melanoma had decreased DPPIV activity in serum, while only 4 melanoma patients had increased DPPIV serum activity (Figure 1). Concerning the presence of metastatic disease, 13 out of 27 melanoma patients without metastatic disease had decreased DPPIV activity, while 17 out of 35 melanoma patients with metastases had decreased activity of DPPIV in serum. Enhanced DPPIV serum activity was observed in 3 patients with metastases and only in one melanoma patient without metastatic disease.

On the other hand, only 1 out of 16 persons with vitiligo had diminished DPPIV activity, as well as 6 out of 40 healthy controls. In addition, 4 people with vitiligo and 7 healthy controls were with higher DPPIV activity.

### Expression of CD26 on immunocompetent peripheral white blood cells

**Figure 2:**
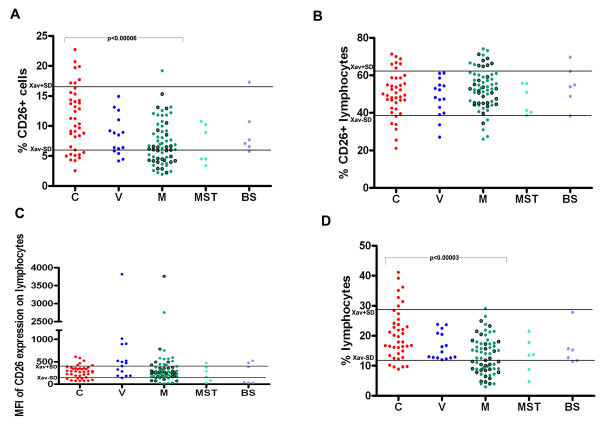
**(A) percentage of CD26+ overall white blood cells, (B) percentage of CD26+ lymphocytes, (C) mean fluorescence intensity (MFI) of CD26 expression on lymphocytes and (D) percentage of lymphocytes, in healthy controls (C), in people with vitiligo (V), in patients with melanoma (M), and in patients with other malignant skin tumors (MST) or benign skin changes (BS).** Xav+/−SD are cut off values obtained from healthy controls. Green circles with borders represent melanoma patients without metastases, while green circles without borders represent melanoma patients with metastatic disease. There is a statistically significant decrease in the percentage of CD26+ overall white blood cells, (Wilcoxon rank sum test with continuity correction: W = 679.5; p < 0.00006), as well as in the percentage of lymphocytes (Wilcoxon rank sum test with continuity correction: W = 644.5; p < 0.00003) in patients with melanoma compared to healthy controls.

Regarding the expression levels of DPPIV (as CD26) on lymphocytes, no significant differences in the percentages of CD26+ lymphocytes (Figure 2B), and in the values for mean fluorescence intensity (MFI) of CD26 expression on lymphocytes (Figure 2C) were found between the melanoma patients, people with vitiligo and healthy control subjects. However, there is a statistically significant decrease in the percentage of CD26+ overall (white peripheral blood) cells in patients with melanoma in relation to healthy control people (p < 0.00006) as seen in Table 1 and on Figure 2A. In the group of 6 patients with other malignant skin tumors decrease in the percentage of CD26+ overall cells was observed when compared to healthy controls, although this difference had not statistical significance (Wilcoxon rank sum test: W = 61; p = 0.055). Furthermore, a higher frequency of patients with melanoma showed decreased percentages of CD26+ overall cells (26 out of 64, cut-off point 6.00% (Xav-SD), as seen on Figure 2A.

Since lymphocyte pool is considered as one of the main sources of soluble DPPIV in serum, the percentages of lymphocytes in examined groups were determined too. It is very important to stress that there is a statistically significant decrease in the percentage of lymphocytes in patients with melanoma in comparison to healthy controls (p < 0.00003), (Table 1 and Figure 2D). Group of people with vitiligo had higher lymphocyte percentage than the group of patients with melanoma, but this difference was not significant (Wilcoxon rank sum test with continuity correction: W = 321; p = 0.048). Additionally, 29 out of 64 patients with melanoma had decreased percentage of lymphocytes, in contrast to 5 out of 40 healthy controls and none of people with vitiligo with lower lymphocyte percentage. The cut-off values for the percentage of lymphocytes were 11.86% (Xav-SD) and 28.92% (Xav + SD), previously obtained from 41 healthy control persons, including 40 involved in this study. On the other hand, 15 out of 64 patients with melanoma had increased MFI of CD26 lymphocytes expression and among 15 people with vitiligo, 8 had increased MFI of CD26 expression on lymphocytes.

### Relationship between the serum DPPIV activity and CD26 expression

It should be mentioned that higher frequency of melanoma patients without metastatic disease had decreased percentage of CD26+ overall cells (14 out of 27) and decreased percentage of lymphocytes (16 out of 27) compared to patients with metastatic disease (frequencies: 12 out of 37, and 13 out of 37, respectively). It is interesting to note that 14 out of 30 melanoma patients who had decreased serum DPPIV activity also had decreased percentage of CD26+ overall cells. There were 15 melanoma patients out of 30 who had decreased DPPIV serum activity and lymphocyte percentage as well. However, 6 out of 30 melanoma patients who had decreased DPPIV serum activity showed an increased percentage of CD26+ lymphocytes. Furthermore, 18 out of 26 melanoma patients who had decreased percentage of CD26+ overall cells had reduced percentage of lymphocytes.

In contrast, only one person with vitiligo, disease with autoimmune etiology, with decreased serum DPPIV activity, had decreased percentage of CD26+ lymphocytes and percentage of CD26+ overall cells, while percentage of lymphocytes was in the range of reference values. In addition, 2 out of 8 people with vitiligo with increased MFI of CD26 expression on lymphocytes showed increased DPPIV serum activity. Moreover, from the 15 out of 64 patients with melanoma with increased MFI of CD26 lymphocytes expression, 6 showed decreased DPPIV activity in serum.

## Discussion

Multifunctional regulatory molecule, dipeptidyl peptidase IV plays an important role in tumorigenesis. Depending on the interaction with specific biomolecules, implicated in initiation, promotion and progression of cancer, and depending on the modulation of bioactive peptides according to the intensity of expression, DPPIV may exert tumor-suppressing activity, as well as completely opposite - tumor-promoting activity [2,4,5].

In the initial steps of pathogenesis of melanoma the alterations in expression and activity of DPPIV might be significant: a) During malignant transformation of melanocytes into melanoma cells, down-regulation of DPPIV expression and later complete loss of expression occurs [13,14,17]; b) DPPIV gene is localized in chromosome 2q24.2, interestingly this chromosome is altered in 35% of melanoma specimens, indicating that loss of DPPIV expression caused by deletion may be even related to progression of melanoma; c) Induction of normal levels of DPPIV expression in human melanoma cells transfected with gene coding for the DPPIV caused loss of characteristics of malignant cells and phenotype changes, suggesting the tumor – suppressing properties of DPPIV [17]; d) Results from one study showed decline in an invasive potential of melanoma cell lines in which DPPIV was reexpressed at levels similar to those in non-transformed melanocytes [15]; e) Additionally, DPPIV was identified as one of the potential markers for distinction between benign deep penetrating nevi and melanoma [16].

Due to its role in regulation of immune, inflammatory and neuroendocrine processes, disturbances in serum DPPIV activity and/or expression are related to different pathophysiological conditions [2,21-27]. For example, lower activity of DPPIV in serum is found in patients with diabetes mellitus, hypertension associated with angioedema induced by angiotensin-converting enzyme treatment, in disorders such as anxiety and depression, in patients with autoimmune diseases and different immune-mediated disorders, including rheumatoid arthritis, systemic lupus erythematosus and inflammatory bowel disease. On the other hand, increase in DPPIV serum activity is related to liver disorders, osteoporosis, infectious diseases and anorexia and bulimia. All mentioned details concerning the complexity of DPPIV should be taken into account for better understanding of its functions. Although it is suggested that activity of DPPIV in serum may decrease with age due to enhanced sialylation [2,3], absence of significant age influence on serum DPPIV activity was documented as well [28,29]. In the group of healthy control persons, no significant age-related differences in DPPIV serum activity were noticed; 3 out of 6 healthy controls who had decreased DPPIV activity were under the age of 30. It is important to note that significant differences between obtained reference values for serum DPPIV activity for healthy persons could be seen in the literature, depending on the experimental procedure and the number of examined subjects. Considering the cut-off values for DPPIV activity in serum, obtained by analyzing 40 healthy control people, our results are in agreement with some of the previously reported data, while at the same time are in contrast to higher or lower values presented in other research articles [2] and references cited therein, [29-32].

We report for the first time that there is a statistically significant decrease in the serum DPPIV activity, in the percentage of CD26+ overall white blood cells and in the lymphocyte percentage in patients with melanoma in comparison to healthy control people. Apparently, decrease in the mentioned parameters observed in the group of patients with melanoma is not dependent on the presence of metastatic disease, since differences in measured parameters were not statistically significant between the subgroup of melanoma patients without metastatic disease and the subgroup of patients with metastatic disease. Significant decline in serum DPPIV activity found in melanoma patients compared to healthy controls might indicate its possible role in development and progression of melanoma, but further research needs to be done in order to fully elucidate the cause and the importance of observed changes in DPPIV activity. However, alterations in DPPIV serum activity observed in the group of melanoma patients might be, at least to some extent, attributed to other individually specific physiological and pathophysiological processes.

Decreased percentage of lymphocytes found in 29 out of 64 examined patients with melanoma points to impaired immune functions, which may affect the course of this malignant disease. In this regard, one of the approaches in the treatment of different forms of cancer is immunotherapy, based on the use of immunomodulatory factors in order to enhance the antitumor immune response, and those agents may elevate level of DPPIV as well.

Changes in the DPPIV serum activity in patients with melanoma associated with decrease in the percentage of CD26+ white blood cells could be explained by significant decrease in the lymphocyte percentage, caused by tumor-specific immunosuppression or by alterations in lymphocytes homing induced by changes in chemokine gradients. Since cell surface CD26 associates with extracellular ADA, changes in CD26 expression on lymphocytes might, at least in some part, influence their overall number as a result of fact that ecto-ADA free lymphocytes are not capable to catalyze and inactivate potentially toxic adenosine to non-toxic inosine [1-3]. However, one explanation to be investigated could be related to changes in the lymphocyte subsets, some richer than another in CD26 expression.

## Conclusions

Results from the present study show for the first time statistically significant decrease in the serum DPPIV activity, in the percentage of CD26+ overall white blood cells and in the lymphocytes percentage in patients with melanoma in comparison to healthy control people. In addition, melanoma patients had significantly lower levels of DPPIV serum activity than people with vitiligo. Obtained data support the relationship between circulating DPPIV and the immune system and may suggest the potential role of DPPIV in complex molecular mechanisms of melanoma pathogenesis. This study indicates the need for exploring the cause and the importance of the disturbances in the serum DPPIV activity and in the CD26 expression on white blood cells in melanoma.

## Competing interests

The authors declare that they have no competing interests.

## Authors’ contributions

IM performed determination of DPPIV serum activity and flow cytometry analyses, interpreted obtained data and wrote the first and last version of the manuscript. MĐ, NG, AD and BK have done flow cytometry and analyzed data. AEN helped with analysis of melanoma patients data. RDž, MŠ and SN enrolled patients with melanoma in the study and interpreted obtained data. DD, SR and SA enrolled people with vitiligo in the study and interpreted data. DG have done statistical analyses. OC helped with data analysis and critically revised the manuscript. ZJ designed the study, interpreted data, participated in writing the manuscript and critically revised the manuscript. All authors have read and approved the final version of the manuscript.
